# Differential processing of intrinsic vs. extrinsic coordinates in wrist movement: connectivity and chronometry perspectives

**DOI:** 10.3389/fninf.2023.1199862

**Published:** 2023-07-10

**Authors:** Laura Alejandra Martinez-Tejada, Yuji Imakura, Ying-Tung Cho, Ludovico Minati, Natsue Yoshimura

**Affiliations:** ^1^School of Computing, Tokyo Institute of Technology, Yokohama, Japan; ^2^School of Engineering, Tokyo Institute of Technology, Yokohama, Japan; ^3^Institute of Innovative Research, Tokyo Institute of Technology, Yokohama, Japan; ^4^Center for Mind/Brain Sciences (CIMeC), University of Trento, Mattarello, Italy; ^5^Neural Information Analysis Laboratories, ATR, Kyoto, Japan

**Keywords:** effective functional connectivity, motor coordinate frames, multivariate pattern analysis, reaction time, wrist movement

## Abstract

This study explores brain-network differences between the intrinsic and extrinsic motor coordinate frames. A connectivity model showing the coordinate frames difference was obtained using brain fMRI data of right wrist isometric flexions and extensions movements, performed in two forearm postures. The connectivity model was calculated by machine-learning-based neural representation and effective functional connectivity using psychophysiological interaction and dynamic causal modeling analyses. The model indicated the network difference wherein the inferior parietal lobule receives extrinsic information from the rostral lingual gyrus through the superior parietal lobule and transmits intrinsic information to the Handknob, whereas extrinsic information is transmitted to the Handknob directly from the rostral lingual gyrus. A behavioral experiment provided further evidence on the difference between motor coordinate frames showing onset timing delay of muscle activity of intrinsic coordinate-directed wrist movement compared to extrinsic one. These results suggest that, if the movement is externally directed, intrinsic coordinate system information is bypassed to reach the primary motor area.

## 1. Introduction

How does the brain enable the body to interact with external physical objects given the enormous multitude of possible body motor coordinate frames? This is a question that neuroscientists have been exploring aiming to find where in the brain this control operates. A usual method to approach this question is to examine the neural representations of the motor coordinate frames. When interacting with the external environment, representing the geometrical coordinates of objects in the outside world is necessary to plan one’s movement with respect to the geometrical coordinates of one’s own body. Since the external and internal body’s coordinate frames exist independently, the brain needs to seamlessly transform between these extrinsic (i.e., Cartesian) and intrinsic (i.e., body or muscle-centered) coordinate frames. Thus far, which brain regions and circuits perform this operation remains unclear.

Studies addressing this question have focused on the motor-related areas in primates using neurophysiological recordings (Evarts, 1968; Cheney et al., 1985; Georgopoulos et al., 1986; Donoghue et al., 1992; Kurata, 1993; Scott and Kalaska, 1995; Kakei et al., 1999, 2001; Pesaran et al., 2006). Furthermore, human studies have covered a range of topographical mapping modalities, including functional magnetic resonance imaging (fMRI) (Eisenberg et al., 2010; Toxopeus et al., 2011; Yoshimura et al., 2014), transcranial magnetic stimulation (TMS) (Davare et al., 2006, 2009; Dafotakis et al., 2008; Alaerts et al., 2009; Duque et al., 2012; Stadler et al., 2012), and positron emission tomography (PET) (Stephan et al., 1995). The primary motor cortex (M1) has been shown to represent both intrinsic and extrinsic coordinate frames, initially in primates and later also in human studies. Intrinsic information such as muscle tension in monkeys (Evarts, 1968; Cheney et al., 1985; Donoghue et al., 1992; Kakei et al., 1999), human muscle-specific resonating activity (Alaerts et al., 2009; Yoshimura et al., 2014), and monkey joint angle (Scott and Kalaska, 1995), were found to be represented in M1. In contrast, some studies in monkeys (Georgopoulos et al., 1986; Kakei et al., 1999) and humans (Eisenberg et al., 2010; Toxopeus et al., 2011; Yoshimura et al., 2014) have additionally shown that M1 also encodes movement in the extrinsic coordinate system.

Another motor-related area, the premotor cortex (PM), represents the extrinsic coordinate system. The ventral region of the PM (PMv) encodes the direction of action (Kakei et al., 2001), while its dorsal part (PMd) encodes motor preparation (Kurata, 1993) and the relative position of targets during reaching in primates (Pesaran et al., 2006). In humans, fMRI and TMS studies have shown the involvement of PMd in representing movement direction (Yoshimura et al., 2014), motor preparation (Davare et al., 2006), and action prediction (Duque et al., 2012; Stadler et al., 2012). On the other hand, fMRI results indicate that the PMv is implicated in representing the motor direction (Yoshimura et al., 2014), alongside motor imagery, motor preparation, and grip force prediction, as shown by TMS and PET studies (Stephan et al., 1995; Dafotakis et al., 2008; Davare et al., 2009). Although the supplementary motor area (SMA) proper and the pre-SMA form parts of PM, the representation of the coordinate system in these areas has not been explicitly examined, except by one fMRI study (Yoshimura et al., 2014). According to that study, the SMA proper represents the direction of movements (i.e., extrinsic coordinate system), while the pre-SMA seems to respond similarly to both coordinate systems.

When addressing other than motor-related areas to study how the brain process information to allow the body’s interaction with physical objects represented in internal and external motor coordinates, previous researches have studied areas related to sensorimotor transformation or visually-guided movements. In this regard, the involvement of the parietal cortex has long been noted, as reflected in the two-stream hypothesis (Jannerod, 1981; Binkofski and Buxbaum, 2013). This hypothesis posits that the dorsoventral and dorsomedial streams mediate the grasping and reaching processes, respectively (Jeannerod, 1999). In primate studies, the dorsomedial stream extends from the primary visual cortex to the medial intraparietal area (MIP) and PMd, while the dorsoventral stream goes to the anterior intraparietal area (AIP) and PMv (Matelli et al., 1986; Tanné et al., 1995; Shipp et al., 1998; Tanné-Gariépy et al., 2002; Borra et al., 2008; Gamberini et al., 2009, 2020; Bakola et al., 2010, 2017; Passarelli et al., 2011). A human study examining the neural representation of motor coordinate frames using a reaching task focused on the posterior parietal cortex (PPC) supports this hypothesis and demonstrates the involvement of the PPC in the extrinsic motor coordinate frame (Fujiwara et al., 2017). Also, an fMRI study focused on the PPC to disentangle the fronto-parietal networks mediating in visuomotor functions during the execution of saccades, hand, and foot pointing, described a functional distinction between lateral region in the posterior intraparietal sulcus (lpIPS), preferring saccades over pointing and coupled with the frontal eye fields (FEF) at rest, and a more medial portion (mpIPS) intrinsically correlated to the PMd (Bencivenga et al., 2023a). On the other hand, a recent high-resolution 7 T fMRI study found that information can be accessed through shared functional connectivity, including the superior frontal and precentral gyrus, central sulcus, intraparietal sulcus, precuneus, and insular cortex (Greulich et al., 2020). Therefore, to examine the neural representations of the motor coordinate frames during motor tasks other than grasping and reaching, it is worthwhile and necessary to probe the entire cortex. Considering the entire cortex can lead to identifying the effective connectivity across brain regions that might transform information between the intrinsic and extrinsic motor coordinate frames, which has not been investigated previously. If there are differences in connectivity between the two coordinate frames, examining whether the differences are related to behavioral data may also help elucidate the mechanisms of motor control.

In this study, we report a representation analysis based on multivariate pattern analysis (MVPA) using fMRI whole-brain data acquired during visually-guided wrist movements performed in two different right-forearm postures. Focusing on the brain regions that the MVPA showed to be predominantly tracking the intrinsic or extrinsic coordinate frames, we conduct a psychophysiological interaction (PPI) analysis to formulate a model regarding which regions receive or transmit intrinsic and extrinsic coordinate frame information in the task. The model was further evaluated and adjusted via dynamic causal modeling (DCM) analysis. To provide further evidence on the information processing path differences between motor activity in the external and internal coordinate frames, we conducted a behavioral experiment examining the reaction times (RT) of four wrist movements performed in three right forearm postures for the intrinsic and extrinsic coordinate frames.

## 2. Materials and methods

### 2.1. Participants

From the original experiment (Yoshimura et al., 2014), participants fMRI data was used for the current analysis, 10 right-handed healthy human participants (2 female and 8 male), between 21 and 47 years old (MD = 34.1, SD = 10.7). In the behavioral experiment, 20 right-handed human participants (7 females and 13 males), between 21 and 51 years old (MD = 29.7, standard deviation SD = 6.2) participated. Written informed consent was obtained from all participants before both experiments. The experimental protocols were approved by the ethics committee of the National Center of Neurology and Psychiatry and the Tokyo Institute of Technology (No. 2022047, 2022).

### 2.2. fMRI dissociable dataset for intrinsic and extrinsic coordinate frames

**FIGURE 1:**
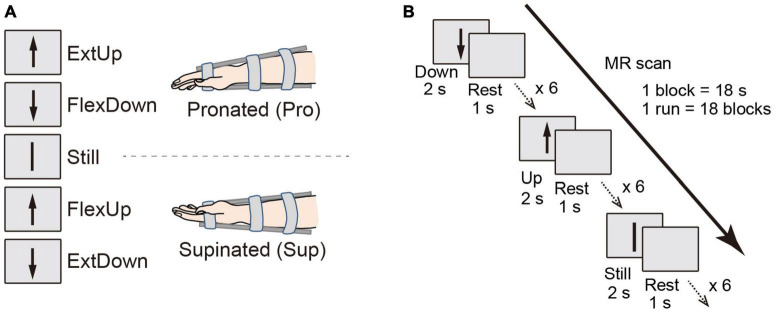
**(A)** Schematic of participant postures and relation between movement directions and tasks according to visual instructions in two different wrist postures. **(B)** Block design for the fMRI experiment.

The fMRI experimental design allows for dissociating coordinate frame information into intrinsic and extrinsic (Yoshimura et al., 2014). Specifically, visual cues for the right wrist flexion (Flex) and extension (Ext) movements were provided by graphical arrows pointing up and down, which can trigger motor commands in an extrinsic coordinate frame manner. One arrow direction cued multiple tasks depending on the wrist posture by changing the forearm postures of the right wrist: pronated (Pro; palm downward), and supinated (Sup; palm upward) (Figure 1A). Table 1 shows the paired data based on arrow directions for the binary classification, the PPI and the DCM analysis.

**TABLE 1:** Combination of the four-condition (ExtUp, FlexUp, FlexDown, and ExtDown) used in the three analyses, binary classification, PPI, and DCM analyses.

**Two types of binary classification analysis**		
FvE	(FlexUp + FlexDown) vs. (ExtUp + ExtDown)	
UvD	(FlexUp + ExtUp) vs. (FlexDown + ExtDown)	
**Two dataset combinations for contrast settings in PPI**		
Intrinsic	Flex Ext	FlexUp + FlexDown ExtUp + ExtDow
Extrinsic	Up Down	FlexUp + ExtUp FlexDown + ExtDown
**Four task settings for DCM analysis**		
Intrinsic	Flex	FlexUp + FlexDown
	Ext	ExtUp + ExtDow
Extrinsic	Up	FlexUp + ExtUp
	Down	FlexDown + ExtDown

All of these combinations were defined according to the analysis method to separate information according to intrinsic and extrinsic coordinate frames.

There were eighteen 18 s task blocks in one functional run (Flex, Ext, and Still, 6 times each; Figure 1B), with a 3 s rest period between the task blocks. The three task blocks appeared in pseudo-randomized order to assure that all the tasks were performed within three consecutive blocks. According to a visual cue of a graphical arrow toward up or down shown on a computer screen, the participants repeated a task (i.e., force exertion or still) six times during the task period, with each exertion lasting 2 s interspersed with 1 s rest periods. A detailed description can be found in Yoshimura et al. (2014).

### 2.3. Data acquisition

A 3 T Magnetom Trio MRI scanner with an 8-channel array coil (Siemens, Erlangen, Germany) was used for the fMRI experiment. Functional data were acquired with a T2*-weighted gradient-echo, echo planar imaging sequence using the following parameters: repetition time (TR) = 3 s; echo time (TE) = 30 ms; flip angle (FA) = 90°; field of view (FOV) = 192 × 192 mm; matrix size = 64 × 64; 36 slices; slice thickness = 3 mm; 140 volumes. The following MP-RAGE T1-weighted sequence was used for a 3D anatomical image (TR = 2 s; TE = 4.38 ms; FA = 8°; FOV = 192 × 192 mm; matrix size = 192 × 192; 160 slices; slice thickness = 1 mm). EMG signals were also recorded using the Delsys Trigno wireless system (Delsys Inc., Natick, MA, USA), and mean muscle activity levels were compared across conditions to determine the consistency of force and muscle activity levels across conditions after the experiment.

### 2.4. Data preprocessing

Functional magnetic resonance imaging data were preprocessed using SPM12 (The Wellcome Centre for Human Neuroimaging, 1991), running on MATLAB R2020b (The MathWorks, Inc., Natick, MA). The preprocessing flow for the classification analysis (i.e., MVPA) differed from the one used for the effective connectivity analyses (i.e., PPI and DCM).

For the classification analysis, all functional images and the T1-weighted anatomical image were realigned and co-registered to the mean image of the functional images, respectively, to keep the voxel values in the functional images unchanged as much as possible. The co-registered T1-weighted image was used to obtain an inverse-normalization transformation matrix to convert region of interest (ROI) masks (described in Section “2.5. Region of interest mask”) defined in the standard Montreal Neurological Institute (MNI) space into individual participants’ native brain spaces. No spatial smoothing was applied to the functional images at this stage.

For the PPI and DCM analyses, on the other hand, we followed the standard preprocessing flow: All functional images were processed with slice-timing corrections, realigned to the mean image of the functional images, and then co-registered to the T1-weighted anatomical image. The co-registered functional images were further normalized to the MNI standard brain space and spatially smoothed with a Gaussian kernel having 8 mm full-width at half-maximum.

### 2.5. Region of interest mask

We used ROIs based on Brainnetome Atlas (Brainnetome Center Institute of Automation, Chinese Academy of Sciences, 2014; Fan et al., 2016) for the classification analysis to cover the whole brain, and the left Handknob [i.e., a sphere ROI with a center coordinate of [−34, −25, 57] (Davare et al., 2010)] the Human Motor Area Template (HMAT) (Mayka et al., 2006) was additionally used for the PPI and DCM analyses. The Brainnetome Atlas divided the whole brain into 246 brain areas, whereas the HMAT consists of 12 motor-related areas; left and right hemispheres of the primary motor area (M1), the primary sensory area (S1), ventral and dorsal premotor areas (PMv and PMd), supplementary motor area (SMA), and pre-SMA.

### 2.6. Binary classification for coordinate frames’ neural representation analysis

We chose MVPA as a neural representation analysis because the method has been recognized to be sensitive to experimental manipulation and areal dissociation in previous studies (Mourão-Miranda et al., 2005; Kriegeskorte, 2011). In our previous study, we have successfully used the method to obtain physiological findings comparable to those obtained with animals. We applied the same classification method, sparse logistic regression (SLR) (Yamashita et al., 2008), as used in our previous study (Yoshimura et al., 2014). Using voxel data included in each ROI, we trained two types of binary classifiers, Flex vs. Ext (FvE) classification and Up vs. Down (UvD) classification, and compared across-participant mean classification accuracies of the two classifiers for each ROI. The idea is that the brain regions representing intrinsic coordinate frame information should show significantly higher classification accuracy in the FvE classification than in the UvD classification. In contrast, the regions representing extrinsic coordinate frame information should show significantly higher accuracy in the UvD classification. The validity of the idea has been proven in our previous study, which showed that the neural representations focusing on motor-related areas were consistent with existing electrophysiological studies of primates (Yoshimura et al., 2014). The target regions were expanded to the whole brain in this study.

The classification analyses were performed for the 246 ROIs separately using images in participants’ individual native spaces. The time series functional data of individual ROIs were extracted from the preprocessed data at six-time points per block, providing 36 scans for each task. To remove temporal baseline shift, mean signal intensity calculated from the 6 scans of the Still task block was subtracted from the signal intensities of the Flex and Ext block data, which can minimize dependency among blocks rather than high-pass filtering used in the standard preprocessing method. The classifiers were trained based on L1-norm based SLR with Laplace approximation using SLR Toolbox version 1.2.1 alpha (Yamashita et al., 2008; Advanced Telecommunications Research Institute International, Japan, 2009) using six-fold leave-one-block-out cross-validation. Specifically, five blocks from each task (20 in total) were used to train a classifier, and the one remaining block from each task (four in total) was used to evaluate the performance of the trained classifier. This was repeated six-fold, with each fold using a unique partition of training and testing blocks.

For each ROI, the mean accuracies for the UvD and FvE classification were first calculated based on each participant’s mean accuracy from six-fold cross-validation. Then, statistical significance comparing the accuracies between UvD and FvE was evaluated by *t*-test using the mean accuracies from all participants.

### 2.7. PPI analysis of effective connectivity

Psychophysiological interaction analysis was performed to reveal the brain areas that show stronger effective connectivity during intrinsic and extrinsic movement tasks. We followed the standard process of the PPI analysis (O’Reilly et al., 2012), but two general linear models were separately estimated using intrinsic dataset and extrinsic dataset combinations (The middle plane in Table 3). The intrinsic combination dataset consisted of flexion tasks (i.e., FlexUp and FlexDown) and extension tasks (i.e., ExtUp and ExtDown), whereas the extrinsic combination dataset consisted of up tasks (i.e., FlexUp and ExtUp) and down tasks (i.e., FlexDown and ExtDown). Time-series data of each seed-ROI was extracted from the individual combination datasets, and voxels showing significant psychophysiological interaction were estimated on contrasts of flexion vs. extension and up vs. down, respectively. Group analysis was performed to identify significant voxels for individual combination datasets.

### 2.8. PPI results model’s validation using DCM analysis

Based on the results from the PPI analysis, we formulated a model representing the differences between the effective connectivity activated with intrinsic and extrinsic conditions. Then, the model was validated using dynamic causal modeling (DCM) analysis implemented in SPM12. We performed the following standard DCM analysis flow (Stephan et al., 2010), but the analysis was repeated 4 times using the different task combinations of the dataset (the lower plane in Table 1). Specifically, time-series data of areas in the model was extracted from the preprocessed functional images, and models to be validated were created for the 4 tasks (i.e., Up, Down, Flex, and Ext) by selecting 2 from the 4 tasks (i.e., FlexUp, FlexDown, ExtUp, and ExtDown). For example, models for the up task were created using FlexUp and ExtUp. Next, the models were evaluated by the Bayesian model selection (BMS) at the group level using fixed-effect analysis (FFX).

### 2.9. Behavioral chronometry of wrist movement

**FIGURE 2:**
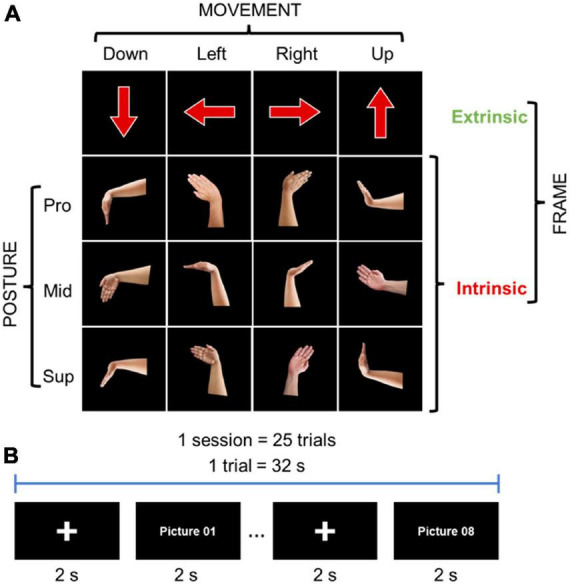
Behavioral chronology experiment method: **(A)** Intrinsic and extrinsic images for visual stimuli: pronation (Pro; palm downward), supination (Sup; palm upward), and midway (Mid; palm leftward). **(B)** Tasks methodology.

Electromyography has proven to be a reliable method for visuomotor RT recordings due to its time resolution and none invasive nature (Tomberg et al., 1991; Ballanger and Boulinguez, 2009). The 20 participants performed 4 wrist movements, flexion, extension, radial deviation, and ulnar deviation, according to visual stimuli. The visual stimuli were provided in an intrinsic or extrinsic coordinate frame manner through an image on a computer monitor. In intrinsic, the images represented hand postures of the 4 movements, while in extrinsic, the images were arrows pointing in 4 directions, up, down, left, and right (Figure 2A). The participants were instructed to perform a wrist movement according to the visual stimulus as fast as possible, and performed the tasks in three sessions by changing the forearm postures of the right wrist: pronated (Pro; palm downward), supinated (Sup; palm upward), and midway (Mid; palm leftward). The tasks were presented 25 times for each task for each posture in randomized order (Figure 2B). EMG signals were recorded using a Delsys Trigno wireless system (Delsys Inc., Natick, MA) at 2 kHz sample frequency. Two electrodes were placed over the right flexor carpi radialis (FCR) and right extensor carpi radialis brevis (ECRB), which are the major muscles for wrist movements. For the pro-down, mid-left, and sup-down movements, the FCR signal was used for analysis, ECRB signal was used for the rest of the movements.

To acquire the EMG wrist movement signal and calculate the RT, the participants were instructed to perform the movement as fast as they could and then go back to a neutral position. The EMG signal was extracted between the 2 s time window of the stimulus presentation. After signal extraction, the mean was removed from the EMG signals and a band pass filter between 20 Hz and 450 Hz was applied, signals were rectified and filtered with a low pass filter at 10 Hz to obtain the EMG envelope. EMG-RT is considered as the time interval between the onset of the time stimulus presentation and the actual onset of the required motor response (premotor and motor time) (Ballanger and Boulinguez, 2009). RTs were calculated from the onset of stimulus presentation to the peak amplitude from the EMG signal within the time window of each picture presentation. Then, RTs were analyzed using a three-way repeated-measures full-factorial ANOVA having Frame, Posture, and Movement as factors. The analysis was performed both on the raw times and on the z-normalized values.

## 3. Results

### 3.1. Neural representation of coordinate frames through comparison of fMRI classification accuracies

In the fMRI experiment, we studied four conditions according to a two-by-two design for the right-wrist movements: an up arrow visual stimulation indicating extension (Ext) in pronated posture (Pro; palm downward) (ExtUp) and flexion (Flex) in supinated posture (Sup; palm upward) (FlexUp), and a down arrow visual stimulation indicating Flex in Pro (FlexDown) and Ext in Sup (ExtDown). This experimental paradigm allows examining the brain activity difference in the two coordinate frames by varying the combination of the four-condition representation (ExtUp, FlexUp, FlexDown, and ExtDown). To elucidate the cortical representation of the extrinsic coordinate system, the data were paired based on the arrow directions: Up data consists of ExtUp and FlexUp, and Down data consists of FlexDown and ExtDown. On the other hand, to elucidate the intrinsic coordinate system, the data were paired based on the movements: Flex data consists of FlexUp and FlexDown, whereas Ext data consists of ExtUp and ExtDown. In this manner, the analyses conducted on such separated datasets enabled examining the distribution of brain activity underlying each of the coordinate systems (Table 1).

**FIGURE 3:**
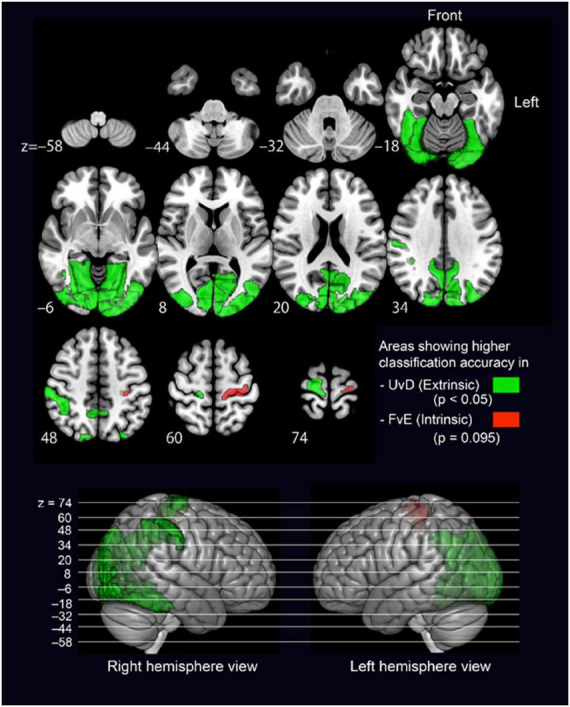
Brain regions showing significantly different accuracy between the UvD and FvE classifications. The upper panel shows axial 2D slices, and the lower panel shows lateral views of 3D brain. Green-colored regions show higher accuracy in UvD, while red-colored regions show higher accuracy in FvE. The *p*-values were calculated using paired *t*-tests.

For the MVPA, we extracted voxel intensity values included in individual anatomical ROIs based on the Brainnetome Atlas (Brainnetome Center Institute of Automation, Chinese Academy of Sciences, 2014; Fan et al., 2016) to cover the whole brain. For each ROI among 246 brain regions, two types of binary classifiers, that is, Flex vs. Ext (FvE) classification and Up vs. Down (UvD) classification, were trained using sparse logistic regression (SLR) (Yamashita et al., 2008). Across-participant average classification accuracies were compared between the FvE and UvD classifiers, and the ROIs showing statistically significant accuracy differences through a paired *t*-test are given in Table 2 and Figure 3.

**TABLE 2:** ROIs showing significant difference between the FvE and UvD classification, the accuracies, and *p*-values.

ROIs with centroid MNI coordinate values (mm)	Classification accuracy (%)		*p*-value
	FvE	UvD	
MVOcC_L54, MedioVentral Occipital Cortex (MVOcC), rostral lingual gyrus (rLinG), [−17, −60, −6]	60.5	**73.8**	*p* < 0.001
LOcC_L44, Lateral Occipital Cortex (LOcC), inferior occipital gyrus (iOccG), [−30, −88, −12]	58.7	**71.9**	*p* < 0.001
PCun_L43, Precuneus, dmPOS, dorsomedial parietooccipital sulcus (PEr), [−12, −67, 25]	56.4	**69.6**	*p* < 0.001
LOcC_L21, Lateral Occipital Cortex, msOccG, medial superior occipital gyrus, [−11, −88, 31]	57.9	**72.5**	*p* < 0.001
MVOcC_L52, rostral cuneus gyrus (rCunG), [−5, −81, 10]	59.9	**75.6**	*p* < 0.001
FuG_L32, Fusiform Gyrus, A37mv, medioventral area37, [−31, −64, −14]	58.4	**70.1**	*p* < 0.001
MVOcC_L55, vmPOS, ventromedial, [−13, −68, 12]	58.8	**72.0**	*p* < 0.001
LOcC_L43, Occipital polar cortex (OPC), [−18, −99, 2]	58.7	**71.7**	*p* < 0.001
MVOcC_L53, Caudal cuneus gyrus (cCunG), [−6, −94, 1]	61.7	**76.7**	*p* < 0.001
MVOcC_R52, Rostral cuneus gyrus (rCunG), [7, −76, 11]	59.9	**74.2**	*p* < 0.001
MVOcC_R54, Rostral lingual gyrus (rLinG), [18, −60, −7]	59.6	**70.7**	*p* < 0.001
LOcC_R21, medial superior occipital gyrus (msOccG), [16, −85, 34]	60.0	**71.2**	*p* < 0.001
LOcC_L41, Middle occipital gyrus (OccG), [−31, −89, 11]	59.3	**73.1**	0.01
MVOcC_R51, Caudal lingual gyrus (cLinG), [10, −85, −9]	59.7	**73.1**	0.03
IPL_L61, Angular, Caudal area 39 (PGp), [−34, −80, 29]	56.6	**62.7**	0.03
IPL_R63, Rostrodorsal area 40 (PFt), [47, −35, 45]	56.3	**65.0**	0.03
LOcC_R44, Inferior occipital gyrus (iOccG), [32, −85, −12]	59.7	**71.2**	0.03
FuG_R32, Medioventral area37, [31, −62, −14]	59.6	**69.8**	0.03
LOcC_R41, Middle occipital gyrus (mOccG), [34, −86, 11]	60.7	**72.0**	0.03
MVOcC_L51, Caudal lingual gyrus (cLinG), [−11, −82, −11]	63.7	**74.2**	0.03
FuG_R33, A37lv, lateroventral area37, [43, −49, −19]	58.7	**67.1**	0.04
PCun_L44, Area 31 (Lc1), [6, −54, 35]	57.5	**64.4**	0.04
LOcC_L42, V5/MT + , area V5/MT + , [−46, −74, 3]	59.8	**66.5**	0.04
MVOcC_R53, Caudal cuneus gyrus (cCunG), [8, −90, 12]	63.7	**75.2**	0.04
PrG_R64, Area 4 (trunk region), [15, −22, 71]	55.4	**62.6**	0.05
PCun_R44, Area 31 (Lc1), [6, −54, 35]	58.2	**65.4**	0.05
PrG_L63, Area 4 (upper limb region), [−26, −25, 63]	**67.4**	62.6	0.09

The bold values are the higher classification accuracies between FvE and UvD binary classification analysis.

**TABLE 3:** ROIs showing significant difference between the FvE and UvD classification, the accuracies, and *p*-values.

Seed	Dataset combination	Target MNI coord. (x, y, z)			Target regions	Cluster-level	
						kE	pFWE
MVOcC_L54, rLinG [−17, −60, −6]	Extrinsic	−30	−24	60	Left PrG	283	<0.001
	Extrinsic	−18	−16	40	White matter	172	<0.001
	Extrinsic	−32	−58	32	White matter	166	<0.001
	Extrinsic	−36	−44	60	Left SPL	172	<0.001
LOcC_L22, occipital gyrus [−22, −77, 36]	Extrinsic	−14	−66	−12	Left Cerebellum Exterior	99	0.008
	Extrinsic	−4	−60	58	Left Precuneus	228	<0.001
>A39c, IPL_L61, Angular [−34, −80, 29]	Extrinsic	−26	−20	48	White matter	173	<0.001
	Extrinsic	34	−10	54	White matter	87	0.02
A39rd, IPL_L62, Angular [−38, −61, 46]	Extrinsic	−42	−28	38	Left PoG	117	0.03
>A39rv, IPL_L65, Angular [−47, −65, 26]	Extrinsic	−46	−8	10	Left central operculum	100	0.043
	Extrinsic	−42	−20	66	Unknown	154	0.006
A37mv, Fug_L32, Fusiform [−31, −64, −14]	Extrinsic	8	−58	54	Right Precuneus	68	0.03
A7r, SPL_L51, [−16, −60, 63]	Extrinsic	−6	−62	50	Left Precuneus	83	0.03
A7pc, SPL_L54, [−22, −47, 65]	Extrinsic	−28	−76	38	Left Angular	197	<0.001
>PoG_L42, [−56, −14, 16]	Extrinsic	50	−4	32	Right PrG	70	0.04
	Extrinsic	−8	−64	38	Left Precuneus	69	0.05
>PoG_L44, [−21, −35, 68]	Extrinsic	−18	−54	−2	White matter	93	0.01
	Extrinsic	10	−50	12	White matter	67	0.05
Handknob, M1 left [−34, −24, 58]	Intrinsic	−28	−72	38	Left Angular	187	0.003
PMv left (HMAT)	Intrinsic	4	−16	16	Right thalamus proper	69	0.016
Pre-SMA left (HMAT)	Extrinsic	−38	−16	62	Left PrG	231	<0.001

All significant regions showed higher accuracies in the UvD classification (i.e., green-colored regions in Figure 3), including the left and right occipital areas (MVOcC), left and right precuneus (PCun), left and right fusiform (Occipitotemporal gyrus), left and right inferior parietal lobules (IPL), and right precentral gyrus, most of which are relating to visual information processing (Chan et al., 2013; Yang et al., 2015). The left precentral gyrus, which is known to represent the intrinsic coordinate frame (Kakei et al., 1999; Yoshimura et al., 2014), showed relatively higher accuracy in the FvE classification, although the effect did not react statistical significance (PrG_L63, *p* = 0.09, red colored region in Figure 3). Altogether, these results suggest that the occipital areas, SPL, fusiform, IPL, and precentral gyrus may relate to neural processing across motor coordinate frames. Therefore, we considered ROIs covering these five regions as seeds for the following PPI analysis.

### 3.2. Psychophysiological interaction analysis

**FIGURE 4:**
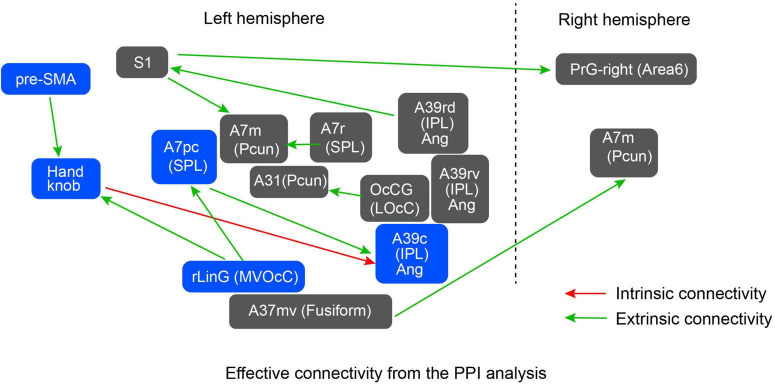
Significant effective connectivity from the PPI analysis. Green arrows denote results obtained using the dataset combination for extrinsic coordinate frame shown in Table 3, whereas the red arrow denotes a connectivity found using the dataset combination for intrinsic coordinate frame. The network consisting of the blue-colored regions was selected as a model to be evaluated by the subsequent DCM analysis.

Psychophysiological interaction analysis was performed to reveal the brain areas showing stronger effective connectivity during intrinsic and extrinsic movement tasks. Since the experimental tasks were performed using the right wrist, we used the five regions in the left hemisphere as seeds for the PPI analysis. Additionally, to examine motor-related areas thoroughly, the following regions of the left hemisphere are also included as seeds: the Handknob, the primary motor area (M1), the primary sensory area (S1), ventral and dorsal premotor areas (PMv and PMd), supplementary motor area (SMA), and pre-SMA. Table 3 shows statistically significant effective connectivity, and Figure 4 summarizes the networks between the significant regions, excluding findings in white matter and basal ganglia. Since the task was performed using the right wrist, most of the connectivity was represented in the left hemisphere. In the intrinsic combination dataset, only one connectivity from the left Handknob to the left Angular gyrus (A39c) reached significance in the left hemisphere. In this study, we aim to elucidate the connectivity difference between the intrinsic and extrinsic motor coordinate frames. Therefore, as shown in Figure 4, we decided to focus on the part of the model encompassing blue-colored regions, namely, the Handknob, IPL (Angular gyrus), MVOcC (rLinG), SPL (A7pc), and pre-SMA for the following DCM analysis.

### 3.3. Dynamic causal modeling analysis

**FIGURE 5:**
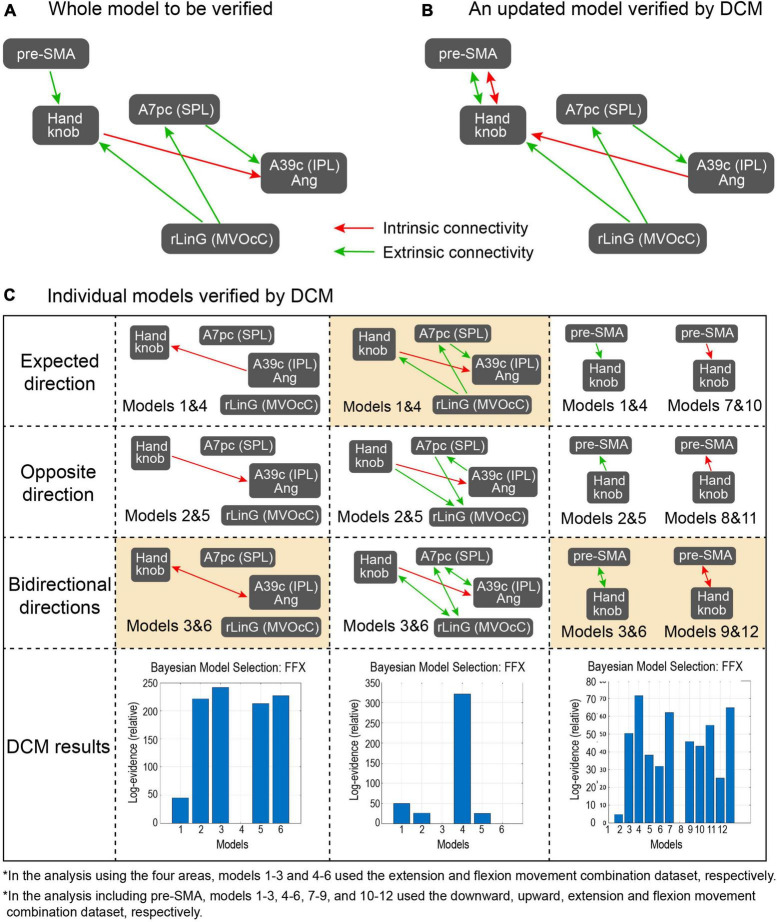
Proposed models and results from the DCM analysis. **(A)** The model proposed from the PPI analysis and to be verified by the DCM, **(B)** The updated model after the DCM, **(C)** Subdivided models that were examined in the DCM analysis. The left, middle, and right columns focus on connectivity in the intrinsic coordinate frame, in the extrinsic coordinate frame, and between pre-SMA and the Handknob, respectively. In each column, models in the first row include expected direction based on the PPI results, those in the second row have connectivity with opposite direction, those in the third row have bidirectional connectivity, and the bottom row shows the probability of the Bayesian model selection done in the DCM. In the models in the left and middle columns, models 1–3 and 4–6 used the extension and flexion movement combination dataset, respectively. On the other hand, in models in the right column, models 1–3, 4–6, 7–9, and 10–12 used the downward, upward, extension, and flexion movement combination dataset, respectively. The orange-colored models showed the highest evidence from the models.

**FIGURE 6:**
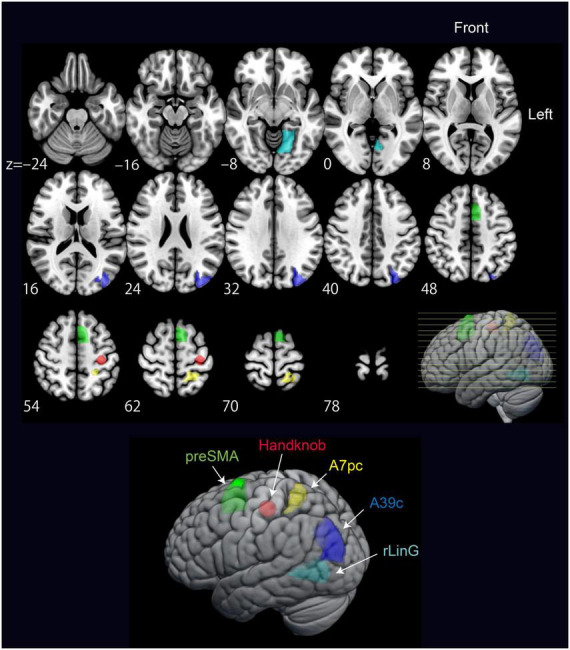
Significant regions included in the model from the PPI results verified by DCM. The regions are pre-supplementary motor area (preSMA), Handknob, IPL Angular gyrus (A39c), SPL (A7pc), and MVOcC (rLinG).

For the DCM analysis, our hypothesis is that model pairs for Flex and Ext, and Up and Down, should show similar tendencies if the fixed-effect defined in the models is promising. Figure 5A shows the model from the PPI results to be verified by DCM, Figure 5B is an updated model based on DCM results, and Figure 5C shows the results of the subdivided models. For the intrinsic connection (i.e., the left column panel in Figure 5C), the models 3 and 6 (3 was from Ext data and 6 was from Flex data) with bidirectional connectivity between the Handknob and the angular gyrus showed stronger evidence than the other unidirectional models. For the extrinsic connections (i.e., the middle column panel in Figure 5C), the originally expected models 1 and 4 (1 was from Down data and 4 was from Up data) with connectivity from the rLinG to the Handknob and SPL, and from the SPL to the angular gyrus showed the highest probabilities than the other models. Finally, for the connection between pre-SMA and the Handknob (i.e., the right column panel in Figure 5C), although the tendencies were not completely identical among the dataset of Down (models 1–3), Up (models 4–6), Ext (models 7–9), and Flex (models 10–12), the bidirectional connections seemed to have the highest evidence for both intrinsic and extrinsic cases. Therefore, the original suggested model in Figure 5A was updated as shown in Figure 5B. On Figure 6 all the significant brain regions included in the model shown in Figure 5B are summarized.

### 3.4. Behavioral chronometry analysis

We hypothesized that, if the model shown in Figure 5B is valid, a temporal delay should occur between executing wrist movements when being instructed in the intrinsic coordinate frame manner as compared to the extrinsic coordinate frame manner. To examine the hypothesis, we performed a behavioral chronometry experiment involving measuring the RTs of wrist movements using electromyography (EMG) from the right forearm [i.e., flexor carpi radialis (FCR) and extensor carpi radialis brevis (ECRB)]. We asked 20 participants to perform four wrist movements: flexion, extension, radial deviation, and ulnar deviation, with three different wrist postures of pronation, supination, and midway, according to visual stimuli showing wrist posture images (i.e., intrinsic coordinate frame manner) or directional arrows (i.e., extrinsic coordinate frame manner, see Figure 2).

**FIGURE 7:**
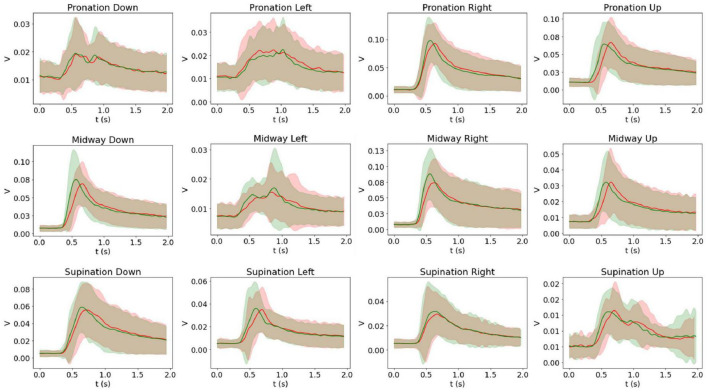
Intrinsic (red) and extrinsic (green) average and standard deviation of the EMG recordings across participants. Twenty participants performed each movement 25 times, and the figures show the grand average across participant averages per each movement. The grand average of the right flexor carpi radialis (FCR) signal is shown for pronation down, midway left, and supination down movements, and the grand average of the right extensor carpi radialis brevis (ECRB) signal is shown for the rest of the movements.

The grand average and corresponding standard deviation of the EMG recording amplitude envelopes across participants are presented in Figure 7. A consistent tendency for signals recorded in response to movements performed under the intrinsic frame to activate and reach their maximum amplitude later than the extrinsic frame homologs is well-evident.

**FIGURE 8:**
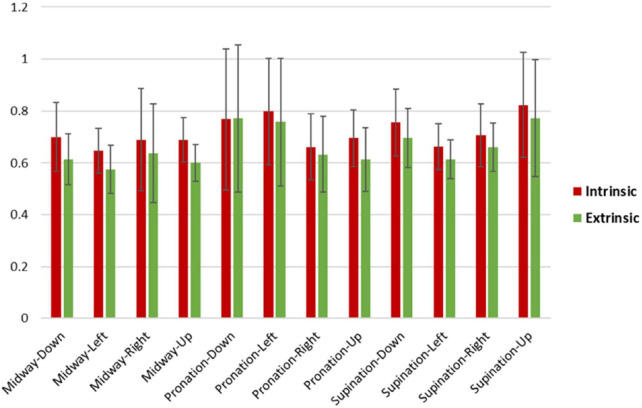
Intrinsic and extrinsic RT means and standard deviations across participants for posture-movement. On average, intrinsic RTs were slower (0.72 s ± 0.06) than extrinsic RTs (0.66 s ± 0.07). Extrinsic midway-left had the fastest RT average (0.57 s), while the slowest RT average was for observed intrinsic supination-up (0.82 s).

As visible in Figure 8, the RTs were consistently longer across postures and movements for the intrinsic than the extrinsic frame. Accordingly, the ANOVA for the raw RTs revealed a strongly significant main effect of Frame [*F*(1,19) = 67.1, *p* < 0.001, η_*p*_^2^ = 0.78] alongside a weaker main effect of Posture [*F*(2,38) = 3.9, *p* = 0.03, η_*p*_^2^ = 0.17], a Frame × Posture interaction [*F*(2,38) = 4.7, *p* = 0.02, η_*p*_^2^ = 0.20] and a Frame × Posture × Movement interaction [*F*(6,114) = 3.0, *p* = 0.01, η_*p*_^2^ = 0.14]. *Post hoc* ANOVAs conducted separately for the three levels of Posture confirmed that the effect of Frame was consistently strongly significant under the pronation [*F*(1,19) = 11.4, *p* = 0.003, η_*p*_^2^ = 0.38], midway [*F*(1,19) = 69.5, *p* < 0.001, η_*p*_^2^ = 0.79] and supination [*F*(1,19) = 33.1, *p* < 0.001, η_*p*_^2^ = 0.64] conditions. The ANOVA for the z-normalized RTs provided analogous results, with a strong main effect of Frame [*F*(1,19) = 49.9, *p* < 0.001, η_*p*_^2^ = 0.72]. The main effect of and interaction with Movement were additionally significant, and in *post hoc* ANOVAs the effect of Frame remained significant across all conditions, not reported for brevity.

## 4. Discussion

In this study, MVPA-based neural representation analysis and effective connectivity analyses inspired the model evaluation positing a difference in effective connectivity between the intrinsic and extrinsic coordinate frames while performing visually-guided wrist movements. According to this model (Figure 5B), signal transmission of the extrinsic coordinate frame information from rLinG would be split into two: one directly to the Handknob and the other via SPL and the IPL (angular gyrus) to the Handknob as intrinsic coordinate information. The model also includes pre-SMA that exchanges the both coordinate information with the Handknob. The model suggests an information transmission difference between the two coordinate frames, which implies that intrinsic coordinate information might arrive to the Handknob later than the extrinsic coordinate information. This aspect of the model was indirectly supported by the behavioral chronometry experiment, which showed longer reaction times of wrist movements visually-guided in the intrinsic coordinate frame compared to extrinsic.

The MVPA-based neural representation analysis across all brain regions revealed that those showing accuracies significant difference between FvE (i.e., intrinsic) and UvD (i.e., extrinsic) classifications were visual processing-related regions, and showed higher accuracy in the extrinsic classification (Table 2; Figure 3). Only the Handknob provided almost significantly higher accuracy in the intrinsic classification; notably, this finding is in line with the results of our previous study (Yoshimura et al., 2014). In our previous study, we showed a voxel-level neural representation by calculating weight values of the individual voxels for the intrinsic and extrinsic classifiers of individual regions and by comparing the mean weight values of the individual regions between the two classifiers. In that way, M1 around the Handknob showed significantly higher weight values in the intrinsic coordinate frame. In our current study, on the other hand, we showed region-level neural representation by simply comparing across-participant mean classification accuracies between the two coordinate frame classifications in accordance with recent conventional practices of machine-learning-based representation analysis (Weaverdyck et al., 2020; Gessell et al., 2021). Therefore, individual differences in voxel-level representation seemed to affect the lack of statistical significance in the Handknob accuracy. Nonetheless, the significantly higher accuracies at low *p*-values of the extrinsic classification in the visual processing related regions (Table 2) suggest the highly plausible representations of extrinsic coordinate frame information in the regions.

Among the significant regions highlighted by the representation analysis, we considered only those in the left hemisphere as the movement task was performed by the right wrist, allowing to simplify the model submitted to the PPI analysis. The assumption to limit the number of regions under consideration, comes from the evidence of interhemispheric inhibition for simple unilateral movements (Ferbert et al., 1992). However, in the case of more complex movements like grasping, it is worth to consider the bilateral involvement of both motor and premotor areas (Bencivenga et al., 2023b).

We also included the seven motor-related areas (i.e., Handknob, M1, S1, PMv, PMd, SMA, and pre-SMA) in the analysis because it is expected that the extrinsic information is transformed to intrinsic information somewhere in the pathway from visual related areas to sensorimotor areas. Most of the PPI results (Table 3; Figure 4) also showed information transmission of the extrinsic coordinate frame, and only one connection between the Handknob and the angular showed intrinsic information transmission. Therefore, we formulated a model mainly consisting of the Handknob and the angular gyrus and evaluated the signal transmission directions by DCM (Figures 5A, B). Since the regions showing significant effective connectivity were included in SPL and IPL, the model formulated in the study does not completely match either stream of the two-stream hypothesis of sensorimotor transformation or visually guided movements. However, it is relatively close to the dorso-ventral stream (Jannerod, 1981; Binkofski and Buxbaum, 2013) as the AIP is included in the supramarginal gyrus (Davare et al., 2010) that has physical connection with the angular gyrus (Seghier, 2013). The wrist movement task used in this study was not reaching nor grasping tasks, but the relative closeness to the dorso-ventral stream of the constructed model might suggest that the task require pathway for grasping rather than reaching.

We employed the behavioral experiment examining reaction times to indirectly evaluate the information transmission difference between the two coordinate frames described by the obtained model. The strongly statistically significant delay in the intrinsic coordinate instruction obtained from the ANOVA should show, at least, the existence of the different pathway between the two coordinate frames, indicating the validity of the network model. RT has long been regarded to represent the functionality of the central nervous system (Lakhani et al., 2012), and it has been found that the reaction time is affected by many factors including age (Welford, 1976; Luchies et al., 2002), anticipation (Welford and Brebner, 1980), arousal (VaezMousavi et al., 2009), stimulus modality (Galton, 1890; Welford and Brebner, 1980), and stimulus intensity (Kohfeld, 1971; Pins and Bonnet, 1996). However, to the best of our knowledge, there are no studies that have examined and significantly revealed differences in reaction time due to neural transmission differences. Also, there are no studies on motor coordinate frames that mention differences in reaction time caused by differences in brain networks, and most of them examine whether components related to movement, such as joints (Scott and Kalaska, 1995), muscles (Evarts, 1968; Cheney et al., 1985; Donoghue et al., 1992; Kakei et al., 1999; Alaerts et al., 2009; Yoshimura et al., 2014), direction (Georgopoulos et al., 1986; Eisenberg et al., 2010; Toxopeus et al., 2011; Yoshimura et al., 2014), force (Saha et al., 2015), and proprioception (Hussian, 2022), are represented in the intrinsic or extrinsic coordinate frames.

Our ultimate interest on the motor coordinate frames is to answer “where in the brain is information between intrinsic and extrinsic coordinate frames during motor control transformed?” To tentatively answer this question, our model indicates that the angular (IPL), Handknob, and pre-SMA seem to deal with both coordinate frame information. However, considering the existence of the two-stream hypothesis, the dorso-ventral and the dorso-medial stream, it is unlikely that the angular is the main area where the transformation takes place, as it is not included in the dorso-medial stream that knowingly mediates grasping process. Alternatively, it might be possible that multiple regions play a role in performing the transformation, which could vary from time to time in a task-dependent manner. To further clarify this question, it would be useful to conduct common analyses of several motor tasks, build a model, and then construct an interventional experimental design that allows evaluation of behavior while function is temporarily blocked by TMS, rTMS or other stimulation methods. While the present work was based on the pre-existing and widely used atlases as Brainnetome Atlas (Brainnetome Center Institute of Automation, Chinese Academy of Sciences, 2014; Fan et al., 2016) and the Human Motor Area Template (HMAT) (Mayka et al., 2006), future work should consider surface-based delineation of the individual ROIs for a more accurate selection of the brain regions.

## 5. Limitations of the study

For the effective functional connectivity analysis, we expected further intrinsic frame dominated brain regions to be identified, but their absence might be partly due to the design of the experiment, where the tasks were instructed by visual stimuli. Likewise, the behavioral chronometry experimental design was not capable of fully examining the model because the intrinsic coordinate movements cannot be induced by visual instruction and we cannot get any anatomical information from the design. In order to fully assess the validity of the model, some sort of intervention methods such as TMS or intracranial stimulation may be useful since there have been arguments on the use of MVPA for representation analysis that might cause misinterpretations (Gessell et al., 2021).

Although the reported findings showed a difference in effective connectivity between the intrinsic and extrinsic coordinate frames while performing visually-guided wrist movements, for the fMRI study, the sample size is relatively small, therefore, the findings should still be considered as preliminary and future work is needed for definite confirmation.

## Data availability statement

The original contributions presented in this study are included in the article/supplementary material, further inquiries can be directed to the corresponding author.

## Ethics statement

The studies involving human participants were reviewed and approved by the National Center of Neurology and Psychiatry and the Tokyo Institute of Technology (No. 2022047, 2022). The patients/participants provided their written informed consent to participate in this study.

## Author contributions

NY: conceptualization. NY and LM: methodology, investigation, and supervision. NY, LM-T, and Y-TC: experiments. YI, LM-T, NY, and LM: formal analysis, writing—original draft, and visualization. YI, LM-T, and NY: resources. LM-T, NY, and LM: writing—review and editing. All authors contributed to the article and approved the submitted version.
